# Life Course Pathways to Later Life Wellbeing: A Comparative Study of the Role of Socio-Economic Position in England and the U.S.

**DOI:** 10.1007/s12062-015-9127-x

**Published:** 2015-07-10

**Authors:** Bram Vanhoutte, James Nazroo

**Affiliations:** Cathie Marsh Institute for Social Research, University of Manchester, Humanities Bridgeford St, M13 9PL Manchester, UK

**Keywords:** Life course, Wellbeing, Comparative, Early life, Social mobility, Accumulation

## Abstract

The influence of early life, accumulation and social mobility on wellbeing in later life in the U.S. and England is investigated. Using cross-sectional data from the Health and Retirement Study (HRS) and the English Longitudinal Study of Ageing (ELSA), we estimate multivariate regressions of hedonic and eudemonic measures of wellbeing on these life course mechanisms, controlling for age, gender, ethnic background, partnership status, health and wealth. On the level of the life course mechanisms, there is mixed evidence regarding the critical impact of early life, strong evidence for an association between accumulation and eudemonic wellbeing and a moderate negative effect of downward social mobility. While the relation between hedonic wellbeing and life course mechanisms is unclear or in a different direction than anticipated, eudemonic wellbeing is clearly related to accumulation and mobility in both countries and to early life in the U.S. On the societal level, the major observation is that the life course has a larger influence in the U.S. than in England.

## Introduction

How do socio-economic trajectories relate to wellbeing in later life in the U.S. and England? A life course view on social background allows us to uncover to what extent appreciations of life quality are shaped by one’s previous experiences. Do people who grew up in relatively poor circumstances appreciate life more than those that had an upper class upbringing? Is it meaningful to think of social position over the life course, as a sum of previous experiences? Does rising or falling in the social hierarchy make a person respectively happier or unhappier? We aim at answering some of these fundamental questions on the relation between wellbeing and life course, by using data from two leading longitudinal surveys of later life, the Health and Retirement Study (HRS), and the English Longitudinal Study of Ageing (ELSA).

### Wellbeing in Later Life

Wellbeing consists of several related but distinct aspects (Deci and Ryan 2006; Diener et al. 1999). Hedonic or subjective wellbeing (Diener 1984), distinguishes moods from cognitive evaluations of one’s life. A second approach, eudemonic or psychological wellbeing (Deci and Ryan 2006; Ryff and Singer 1998), emphasises the role of psychological needs such as autonomy and self-actualisation in wellbeing. This multidimensional nature of wellbeing persists in later life (Vanhoutte 2014). Scrutiny of the evolution and determinants of both forms of wellbeing in later life illustrate different age trajectories as well as different determinants (Jivraj et al. 2014). The cognitive and evaluative aspects of wellbeing are strongly subject to processes of adaptation and changing goals, which is in line with theories of selective optimisation with compensation in later life (Baltes and Baltes 1990): Expectations change over age, and subjective appreciations of quality of life become more positive, although objectively speaking circumstances might be worse. Eudemonic wellbeing on the other hand, uses a stricter, external yardstick for wellbeing: the degree of self-actualisation, autonomy and control, measured subjectively (Ryff and Keyes 1995). The decline of physical health and social support that often accompany ageing, have strong and detrimental effects on these feelings of control and self-actualisation in later life and are hardly affected by adaptation. As such we can expect stronger associations with life course measures for eudemonic than for hedonic wellbeing.

### Socio-Economic Influences over the Life Course

Life course models try to grasp the essence of socio-economic trajectories and how they are linked to both mental and physical health outcomes. A life course approach enables a more dynamic view on socio-economic position, and refines the common assumption that the circumstances at time of measurement reflect one’s position over a lifetime (Hayward and Gorman 2004). Childhood conditions set in motion a complex cascade of direct and indirect influences on both physical and mental health outcomes in adulthood and later life (Ben-shlomo and Kuh 2002; Preston et al. 1998). A parsimonious conceptual overview of possible influences sees four major possible paths between childhood circumstances and adult health outcomes: (1) A direct and negative “scarring” effect, (2) a direct and positive “immunizing” effect, (3) an indirect and negative effect of “correlated environments”, and (4) an indirect and positive “selection” effect (Preston et al. 1998). Three general theories on how socio-economic differences in exposure can be related to health outcomes across the life course summarise these pathways: Critical Period or Latency Model, Accumulation, and Social mobility.

One powerful example of direct childhood influences is the hypothesis that maternal malnutrition during gestation increases the risk of cardiovascular disease and diabetes in adults (Barker 1994). This enduring influence of early life, not limited to fetal development but also encompassing childhood infectious diseases, the socio-economic circumstances and educational level of parents, is more generally framed in a critical period or latency model (Ben-shlomo and Kuh 2002; Haas 2008; Niedzwiedz et al. 2012). In a more sociological sense, the importance of childhood for later life refers to socialisation, or the complicated process of internalising standards, norms and values. These socially inherited norms are relevant as a basic psychological framework for comparison. The concept of generations builds on the stability of these norms, acquired during the adolescent years (Mannheim 1952). In the context of wellbeing this could mean for example that people who grew up in modest circumstances appreciate the general societal rise in living standards over their life time more than people who grew up in wealthy households. Clearly there are theoretical reasons that support a scarring or immunizing early life influence on wellbeing, either through social or biological mechanisms. Although the relevant critical period often depends on the exact phenomenon under study, childhood will be taken as the critical period as this is the dominant approach. The crucial point in this approach is that the early life circumstances set the stage for adult health and wellbeing, and are only marginally affected by later experiences and circumstances.

A second life course model, accumulation, emphasises the indirect nature of early life impact, as a starting point in a sequence of possible exposures to risk factors (Hallqvist et al. 2004; Kuh et al. 2003; Otero-Rodríguez et al. 2011; Singh-Manoux et al. 2004). A person’s biopsychosocial system is affected by insults over the life course, and those in a more disadvantaged social position have to endure more and longer exposure to episodes of injury and illness, adverse environmental conditions and health damaging behaviours. Accumulation is biologically grounded in the concept of allostatic load, or the chronic exposure to fluctuating or heightened neural responses to stressors (McEwen and Stellar 1993; McEwen 2000). A sociological perspective on accumulation is the Matthew effect (Merton 1968), summarised with the tagline that the rich get richer while the poor get poorer. Cumulative (dis)advantage, or the systemic tendency for increasing divergence (Dannefer 2003; Ferraro and Shippee 2009), reconceptualises this as disadvantage increases exposure to risk, advantage increases opportunity. In short, the accumulation model suggests that the length of exposure to adverse socio-economic circumstances is crucial, and that this cumulative effect determines one’s level of wellbeing more than just the current position.

A third life course perspective is social mobility, the process by which people move up or down the social hierarchy across the life course. There are two ways of studying mobility: intra-generational mobility, or the mobility experienced by a group or person over a certain period, and intergenerational mobility, the social change between one generation and another, which we will do. A second important distinction is that we see social mobility in a sociological way, as a change in social position, while an economic perspective equates it with changes in income or in the relative income. It has been established that the direction of change in social hierarchy can have an influence on mortality, in the sense that upwardly mobile have a higher mortality, and downwardly mobile have a lower mortality than those who are stable members of a class (Blane et al. 1999). As social mobility is a form of selection, an inverse influence of health on mobility is a possibility, in the sense that upwardly mobile people are healthier than other class members. As wellbeing is related to expectations, normative framing and adaptation to circumstances, we expect relations to be more complicated than found in studies investigating physical health. It has been illustrated that wellbeing is related to material aspirations, which change as one grows older (Easterlin 2001). By definition, intergenerational social mobility entails that there is some degree of inconsistency in the current and parental social position. This feeling of not entirely belonging, of being between two worlds, can potentially have a negative impact on wellbeing in later life.

There is mixed influence of all three life course models on quality of life and wellbeing (Niedzwiedz et al. 2012). It should also be kept in mind that the different life course models do not necessarily exclude one another, as it is empirically hard to distinguish between them (Hallqvist et al. 2004). Early life can influence later life wellbeing through accumulation, and determine in which direction and to what extent social mobility is possible. As such, we are more weighing the relative importance of each perspective in the context of well-being, rather than accepting or dismissing particular frameworks.

### Comparing Later Life Wellbeing and the Life Course in the U.S. and England

We use a comparative framework to arrive at stronger conclusions regarding the influence of different life course models on later life wellbeing. While a number of comparisons between England and the United States have already been done on causes and early life associations of physical health in old age (Avendano et al. 2009; Banks et al. 2006; 2009), wellbeing has been under less scrutiny. One exception is Jivraj and Nazroo (2014) who found that physical health and wealth are the main determinants of different forms of later life wellbeing in England and the U.S. Socio-economic inequality has a larger influence in wellbeing in the U.S. according to the same study, which suggests that welfare provisions in England, such as public healthcare, manage to attenuate the influence of social inequality. Although wellbeing seems to be produced by similar resources in both countries, there is evidence for a country-specific impact as well.

Some idiosyncratic aspects of each country’s history and development are important to keep in mind in connection with life course concepts.

A first important point is that both countries have different scientific approaches in terms of which markers are used to investigate SEP. Both traditions can be brought back to conceptual differences on the nature of social class grounded in the writings of Marx and Weber (Liberatos et al. 1988). The British tradition relies heavily on the Marxist assumption of occupation as the base of all social differences (see for example (Goldthorpe 2002), while in the U.S. social class is fundamentally multidimensional. In this more Weberian concept of social class, occupation, status (in the form of education) and actual resources (or income) are interrelated and together determine social class (Krieger et al. 1997). It is essential to understand the profoundly different meaning of education in the U.S. before the Second World War. High school education expanded enormously in the U.S., with attendance rates increasing from one out of three in 1920 to one out of two in 1940 (Herbst 1997). In line with this, the average years of schooling increased from seven and a half in 1910 to nine and a half in 1940, while in 1930 in the U.K. on average people had less than 7 years of schooling (Murtin and Viarengo 2009). As such, education in itself becomes an important gateway for distribution of resources in the U.S. fairly early in the 20th century. As it gives access to occupations with more prestige and income, and has large effects on the accumulation of (dis)advantage over the life course (Dannefer 1987), in U.S. research education is often used as a shorthand for social class. In England the focus tends to be on occupational class, and it has been shown that occupational class divisions are more salient in England, both culturally (Gerteis and Savage 1998) and in terms of their health effects (see for example Kunst et al. 1999).

A second important point to think about in our comparison is social mobility. There is a historical and popular conception of the U.S. as a land of exceptional opportunity, a society with little barriers where the “American Dream” of upward social mobility is a reality for those who work hard enough, while England is seen as a rigid class society. This American exceptionalism in terms of social mobility is illusionary, as mobility is slightly higher than in the UK, but comparable to other European countries such as for example Sweden (Erikson and Goldthorpe 1985). In a life course perspective the social mobility of an individual is a reflection of larger societal changes in the structure of the economy. The transition from an industrial to a service economy which happened in both countries at a similar pace in theory accommodates social mobility, but in practice might lead to early retirement or chronic unemployment. Given unemployment rates have been historically higher in the U.K. throughout, but especially in times of transformation, it is possible that the higher social mobility in the U.S. is partly a function of the higher degree of integration of former manual workers in the service industry.

### Research Questions and Hypotheses

Our analysis wishes to investigate the relevance of three life course perspectives, early life, accumulation and social mobility, on both hedonic and eudemonic wellbeing in later life in two countries, England and the U.S. In a more formal framework, this translates in the following research questions and hypotheses:

1. Does early life, accumulation or social mobility explain most of the variance in wellbeing in later life?
  1. The accumulation perspective explains the large share of differences in wellbeing in later life.
2. Are there differences between hedonic and eudemonic wellbeing in the associations with life course mechanisms?
  Hypothesis 2 Eudemonic wellbeing has a closer association with all life course mechanisms than hedonic wellbeing.
3. Do the U.S. and England differ in terms of life course relevance?
  Hypothesis 3 Life course measures have a larger influence in the U.S. compared to England.

## Data and Methods

We will use both the Health and Retirement Study (HRS) and the English Longitudinal Study of Ageing (ELSA). Both studies are panel studies of the community-residing population aged 50+, which refresh their sample regularly to ensure representativity, and conduct core interviews every 2 years. The HRS was first fielded in 1992 and joined with a companion study, the Asset and Health Dynamics of the Oldest Old (AHEAD) in 1998, representing the entire U.S. population age 50+ (*N* = 21,384). Response rates at baseline were 70 to 81 %, and the retention rate for the combined cohorts is 88 %. The ELSA began following a cohort of people aged 50 or older and their partners in 2002. Its sample was drawn from a prior survey, the Health Survey for England (HSE), an annual cross-sectional study that includes an interview and nurse visit (*N* = 12,099). ELSA conducts face-to-face interviews every 2 years. The response rate at baseline was 67 % and the retention rate in wave 5 of wave 1 core member is 66 % (Cheshire et al. 2010). An earlier comparison of HRS and ELSA established that although attrition seems to be a larger problem in ELSA than HRS, this does not affect findings regarding the different prevalence of disease, or the relation between SEP and health in both countries (Banks et al. 2010). A self-completion Leave-Behind Questionnaire (LBQ) was part of ELSA since its first wave in 2002 and identical for all participants. In HRS, two different versions of the LBQ, were administered in 2004 to a pilot sample (*N* ~ 7600 in total, so about *N* ~ 3300 for each version) in 2004. A revised version of this LBQ was administered to half of the HRS sample in 2006, and to the other half in 2008. This questionnaire was administered again to each half of the sample respectively in 2010 and 2012.

### Wellbeing Measures

We make a distinction between two forms of wellbeing, hedonic and eudemonic, respectively measured by the. Our wellbeing measures are part of the LBQ, and to minimise period effects we analyse the same survey years. In line with the current understanding of the conceptual nature of wellbeing, both hedonic and eudemonic wellbeing are investigated, measured by the Satisfaction with life scale (SWLS) (Diener et al. 1985) and CASP (Hyde et al. 2003) respectively (Ryan and Deci 2001). Both scales have good psychometric properties (Vanhoutte and Nazroo 2014), and although they are strongly correlated, reflect different aspects of wellbeing in later life (Vanhoutte 2014). The SWLS has five items (for example “I am satisfied with my life”) that are rated on a 7 point scale ranging from strongly agree to strongly disagree. The measure used to tap eudemonic wellbeing, CASP, consists of 19 items (for example “I feel left out of things”, “I look forward to every day”, I feel that life is full of opportunities”) over three domains (Control and Autonomy, Self-actualisation and Pleasure), that have to be rated on a four point frequency scale ranging from often to never. Here we will examine the revised 15 item version of CASP (Hyde et al. 2003; Vanhoutte 2012) in 2004 (HRS *n* = 3231, Elsa *n* = 8199), and satisfaction with life (Diener et al. 1985) in 2008 (Hrs *n* = 6968, Elsa *n* = 9221). We imputed the scores on these scales if the respondent answered at least half of the items to minimise missingness. The descriptives of both wellbeing scales (Table 1) illustrate that means are very similar across both countries.

**Table 1:** Descriptives of wellbeing measures in Elsa (waves 4 and 2) and HRS (waves 9 and 7)

	England			U.S.		
	N	mean	S.D.	N	mean	S.D.
SWLS (5–35)	9221	25.38	6.23	6968	24.59	7.66
CASP15 (15–60)	8199	49.77	7.29	3231	50.73	7.11

### Life Course Indicators

To operationalise the life course we will make use of comparable information at three time points (early life, early adulthood and late midlife) in both surveys. While we use the occupation of the father when the respondent was 14 in ELSA as early life information, this information is masked in HRS, so that we have to rely on the years of education of mother or father. The educational level of the respondent is used as an indicator of early adulthood. For late midlife, we use the occupational class of the respondent, and rely on the current or last job before retirement in ELSA, and of the longest job in HRS. We miss out on a substantial amount of early life information, as in ELSA, about 20 % classified the job of their father as “other jobs” or “something else”, and in HRS about 13 % did not report the educational level of their mother or father, so we have to exclude these respondents from our analysis. There is a similar proportion of missingness on the HRS longest job variable, measuring occupational class in late midlife.

Table 2 below lists the descriptive statistics of the three measures of socio-economic position at different points in the life course by country in the 2004 waves. The 2008 waves that are used to analyse hedonic wellbeing are essentially the same. To ease comparison, each measure was recoded in three categories. While it is relatively straightforward to recode achieved educational level and occupational class in three categories, matching the number of years of education of parents in HRS to the parental occupational class needs some extra attention and explanation. As we want to be able to draw theoretically robust conclusions on the nature of socio-economic trajectories across the life course in two countries, it is important to have a similar, comparable starting point. Due to the educational expansion in the U.S. in the first half of the 20^th^ century the average number of years of parental schooling was about nine. We use 8 years of education as a cut-off point for low parental education, more than 8 and less than 12 years for a middle level of education and 12 or more years for high levels of education. Although we would have preferred to use the same measure in both countries, the different operationalisation in terms of early life measures clearly has an advantage as well, as they are more country-specific measures of the socio-economic position of one’s parents. Due to these differences in operationalisation, we cannot make exact comparisons on the level of the coefficients, but the comparisons on the conceptual level may capture the life course effects of country-specific markers of social position better.

**Table 2:** Descriptives of life course indicators of socio-economic position in ELSA wave 2 and HRS wave 7

	England (*n* = 8199)		U.S. (*n* = 3231)	
	Measure	%	Measure	%
Early life	Parental class (*n* = 6412)		Parental education (years) (*n* = 2804)	
	Manual	47.9	Low (0–8)	45.7
	Service	17.9	Middle (8.5–11)	19.2
	Professional	34.2	High (12+)	35.1
Early adulthood	Educational level (*n* = 8179)		Educational level (*n* = 3231)	
	Low	35.7	Low	19.8
	Mid	38.7	Mid	37.5
	High	25.6	High	42.8
Late midlife	Occupational class (last/current job) (*n* = 8028)		Occupational class (longest/current job) (*n* = 2877)	
	Manual	41.9	Manual	26.9
	Service	25.1	Service	41.5
	Professional	33.0	Professional	31.6

### Other Variables in the Study

The control variables that we use in this study are listed by country in Table 3, and are based on the respondents that answered the eudemonic wellbeing measure in wave 2 in ELSA and wave 7 in HRS. There are no substantial differences in the descriptive statistics of the control variables with wave 4 in ELSA and wave 9 in HRS, when our hedonic measure of wellbeing was measured. Age and age squared will be included, as they have shown to be important determinants of wellbeing (Jivraj et al. 2014). We also distinguish between partnered (married or cohabited) and unpartnered (single, widowed or divorced). Current health in this study will make use of a functional definition of health, the limitations to (instrumental) activities of daily living (ADL). We used a list of 11 activities present in both studies: all basic ADL (dressing, eating, walking, bathing, getting out of bed, toilet) and some instrumental ADL (preparing a hot meal, handling money, shopping, taking medication, using phone). It has been shown that some items behave differently in ELSA and HRS, but that these differences are mitigated when using the summary scale (Chan et al. 2012). Following this recommendation we dichotomise this measure, and distinguish between having none or at least one limitation in activities of daily living. We include ethnic background, coded as white and non-white, as a control. As African-Americans are oversampled in HRS, this is a necessary condition to come to reliable population estimation. Due to the small numbers of non-white respondents in the English sample, and on the very different history, composition and experience of non-white groups in both populations, we do not make comparisons between countries. A last, but very important aspect we will control for is the level of non-pension household wealth. Since we are interested in the influence of the life course trajectory, rather than current social position, controlling for wealth quintiles allows us to avoid confounding current status with life course trajectory. The main difference between England and the U.S. in terms of our control variables is clearly the larger proportion of older people with a different ethnic background in the U.S.

**Table 3:** Descriptives of control variables by country in ELSA wave 2 and HRS wave 7

	England			U.S.		
	N	mean	S.D.	N	mean	S.D.
Age	8199	65.18	10.28	3212	65.12	10.68
Gender	8199	.56	.50	3231	.59	.49
Partnered	8199	.72	.45	3229	.72	.45
Limitations in (I)ADL (1 or more)	8197	.22	.41	2900	.18	.38
Non-white ethnicity	8197	.02	.15	3231	.12	.28
Wealth quintiles	7559	/	/	3231	/	/

### Methods

An initial descriptive analysis of the prevalence of life course trajectories in each country will give insight into the similarities and differences in life courses in each country. Multivariate linear regression analysis (OLS) will be used to illustrate the direct association between specific life course mechanisms and later life wellbeing. A number of factors entangled with the life course, such as age, gender, ethnic background, partnership status, health (and wealth) are used as controls, and their coefficients are not reported for the sake of brevity. For each life course measure, two different models are presented, a model with and a model without current wealth in the controls. Both models have their value in understanding the importance of life course measures for wellbeing: while a model without controlling for wealth illustrates a more general effect of socio-economic life course trajectories, controlling for wealth investigated to what extent the life course effect operates over and above the current status. The increase in explained variance by adding the life course measure to the analysis, as well as the total explained variance is reported.

## Analysis: Life Courses Trajectories in the U.S. and England in the Second Half of the 20th Century

To investigate the different life course paradigms, we use life course markers as building blocks to construct meaningful life course measures for these mechanisms. Since each step in the life course at least partly depends on the previous one, it is important to have robust and conceptually defined life course measures. A first important step in our analysis is therefore to descriptively investigate the life course trajectories in our data. Table 4 lists the three most prevalent life course trajectories by point of origin in each country.

**Table 4:** Most prevalent life trajectories by point of origin (% within each group) in ELSA wave 2 and HRS wave 7

	England			U. S.		
Parental status	Educational level	Occupational class	%	Educational level	Occupational class	%
Low education / Manual class England (*N* = 3006) U.S. (*N* = 1153)	Low	Manual	30.0	Low	Manual	14.7
	Middle	Manual	16.5	Middle	Service	20.8
	High	Professional	13.0	High	Professional	17.9
Middle education / Service class England (*N* = 1117) U.S. (*N* = 450)	Low	Manual	15.4	Middle	Service	23.1
	Middle	Service	16.7	High	Service	12.9
	High	Professional	21.6	High	Professional	26.0
High education / Professional class England (*N* = 2146) U.S. (*N* = 909)	Middle	Service	14.4	Middle	Service	13.9
	Middle	Professional	12.8	High	Service	20.9
	High	Professional	30.9	High	Professional	39.9

In the English sample, a third of the people who grew up in a working class family had a low level of education and worked in manual class jobs all their life. This stable low trajectlife satory also exists in the States, but is remarkably less common. The stable high trajectory on the other hand is more prevalent in the U.S. While in England those from a modest background that did finish high school often still ended up in manual class jobs, it is clear that in the States a high school degree functions as a gateway to service class jobs. In both countries higher levels of education function as a means of access to professional class occupations, although more often so for those with a parental higher social background, maybe hinting at social and cultural aspects of social class that are hidden in our analysis, but do play a part in social mobility. While it is not uncommon for the English people in this specific cohort to descend on the social ladder during their lifetime, this is less the case for their U.S. counterparts, among whom upwardly mobile trajectories are more present.

### How Critical is Early Life for Later Life Wellbeing?

The impact of early life is investigated through the socio-economic position of parents. It is important to remember that a significant effect of early life does not necessarily mean that there is a scarring effect on wellbeing by growing up in a family with a working class background or a low level of education. The fact that both in the U.S. and England social origins to a large extent predict one’s own social destination means that the effect of parental SEP could have a largely indirect effect.

We report the results of a regression investigating the relation between parental position and current level of hedonic and eudemonic wellbeing in England and the U.S. in Table 5 below. There is very little consistency in the relation between parental socio-economic position and hedonic wellbeing in both countries. While there is a negative association only in the model controlling for wealth in England, there is a positive association only when not controlling for wealth in the U.S. This negative association in England point to higher discrepancy between expectations and reality in those that grew up in higher socio-economic positions. Given that it only surfaces after taking into account current wealth, it suggests that the needs of those with higher social origins surpass their current means. Eudemonic wellbeing is positively related with parental socio-economic position in both countries, but this association only remains significant when adjusting for current wealth in the US. This suggests that early life influence on eudemonic wellbeing is largely transmitted through wealth in England, but only partially so in the U.S. In sum, parental socio-economic position only has a limited contribution to wellbeing in later life.

**Table 5:** Early life influences on later life wellbeing

	England				U.S.			
	Hedonic (*n* = 6400)		Eudemonic (*n* = 5879)		Hedonic (*n* = 5399)		Eudemonic (*n* = 2517)	
	A	B	A	B	A	B	A	B
	B (SE) P	B (SE) P	B (SE) P	B (SE) P	B (SE) P	B (SE) P	B (SE) P	B (SE) P
Early life SEP (ref. Low)								
Mid	−.069 (.206) ns	−.333 (.207) ns	.769 (.243)**	.368 (.241) ns	.241 (.281) ns	−.023 (.277) ns	.858 (.354)*	.584 (.347) ns
High	.005 (163) ns	−.428 (.168)*	.585 (.197)**	−.143 (.200) ns	.745 (.226)**	.167 (.227) ns	1.585 (.294)***	1.085 (.291)***
R square	.121	.135	.152	.180	.098	.127	.151	.188
Increase in R square compared to model without	.000	.002	.002	.001	.001	.000	.010	.004

Unstandardized regression coefficients of multivariate regression model (adjusted for (A) age, age^2^, gender, partnered, ethnic background, limitations in ADL and (B) + wealth quintile) *P*=<0.001 ***, *P*=<0.01 **, *P*=<0.05 *, *P*>0.05 ns

### Accumulation

To examine the influence of accumulation, we follow the approach used by Singh-Manoux et al (2004): Summary scores of accumulation are created to reflect the exposure level at the three time points for each trajectory, ranging from 0 (high SEP at three time points) to 6 (low SEP at three time points). Score 1 represents three different trajectories, with one time point in a middle SEP and the other two in a high SEP. Six different trajectories are presented by score 2: three trajectories with a low SEP at one time point and a high SEP at the two others, and three trajectories with a middle SEP at two time points and a high SEP at one time point, etc. In this way each score represents a higher level of exposure than the previous one. Two important assumptions accompany this approach: that the impact of exposure is the same for the three time points, and thowthhat the impact of the difference between a low and a middle SEP is the same as the difference between a middle and a high SEP.

**Fig. 1:**
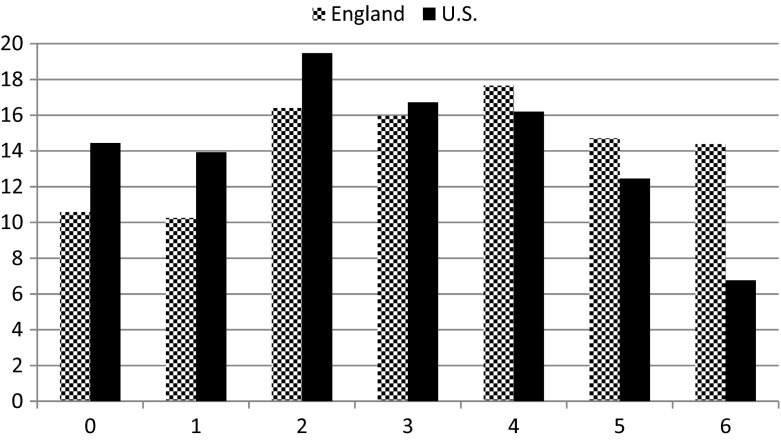
Distribution of accumulation scores (in %) in ELSA wave 2 (*n* = 6,269) and HRS wave 7 (*n* = 2,512) (2004)

Figure 1 gives an overview of the distribution of exposure levels in both England and the U.S. It is evident that there are more people with higher accumulation scores in England, while in the U.S. people with lower scores are more present in the population.

Table 6 lists the results of our multivariate analysis of the influence of accumulation on later life wellbeing. The results of this analysis in both countries are in accordance with each other, and show strong support for an influence of accumulation on eudemonic wellbeing, which remains significant after controlling for current wealth. Accumulation has a graded, dose response, type association with eudemonic wellbeing, and this relation is markedly stronger in the U.S. than it is in England (as shown by the larger differences in the unstandardized coefficient). The relation between accumulation and life satisfaction in the U.S. indicates important differences between lower accumulation levels and the others. In England only the two extreme accumulation profiles (0 and 6) have a significant and positive impact, of which only the highest accumulation category remains significant after controlling for current wealth. This significantly higher level of satisfaction, ceteris paribus, for those who have been in a lower SEP on all three time points is a surprising finding.

**Table 6:** Accumulative influences on later life wellbeing

	England				U.S.			
	Hedonic (*n* = 6188)		Eudemonic (*n* = 5786)		Hedonic (*n* = 4779)		Eudemonic (*n* = 2263)	
	A	B	A	B	A	B	A	B
	B (SE) P	B (SE) P	B (SE) P	B (SE) P	B (SE) P	B (SE) P	B (SE) P	B (SE) P
Accumulation								
0	.925 (281)**	.458 (.284) ns	1.186 (.350) **	.523 (.352) ns	1.953 (.374)***	1.076 (.376)**	2.242 (.463)***	1.490 (.466)**
1	.543 (.281) ns	.192 (.284) ns	.529 (.349) ns	.099 (.349) ns	.812 (.367)*	.256 (.365) ns	1.016 (.471)*	.588 (.468) ns
2	.313 (.253) ns	.143 (.252) ns	.602 (.306) *	.345 (.304) ns	1.113 (.347)**	.678 (.344)*	.257 (.429) ns	−.108 (.425) ns
3 (Ref)								
4	−.090 (.258) ns	.147 (.258) ns	−.554 (.300) ns	−.287 (.298) ns	.025 (.361) ns	.065 (.356) ns	−1.593 (.450)***	−1.541 (.444)**
5	−.159 (.271) ns	.192 (.272) ns	−1.354 (.313)***	−.803 (.314)*	−.083 (.404) ns	.267 (.399) ns	−1.921 (.489)***	−1.525 (.485)**
6	.609 (.296)*	1.219 (.302) ***	−1.492 (.315)**	−.686 (.320)*	−.379 (.496) ns	.266 (.493) ns	−3.069 (.622)***	−2.633 (616)***
R square	.124	.136	.169	.185	.105	.130	.184	.206
Increase in R square compared to model without	.003	.003	.015	.003	.009	.002	.048	.025

Unstandardized regression coefficients of multivariate regression model (adjusted for (A) age, age^2^, gender, partnered, ethnic background, limitations in ADL and (B) + wealth quintile) *P*=<0.001 ***, *P*=<0.01 **, *P*=<0.05 *, *P*>0.05 ns

Two issues may be relevant to this finding. Firstly, the group having the highest accumulation score is almost twice as large in England compared to the U.S. Secondly, comparing scores between the model that controls for wealth and the model that doesn’t, helps us interpret this score. The lowest accumulation score (0) has significantly higher hedonic wellbeing, which can be explained by higher current wealth. The highest accumulation score (6) has a barely significant higher level of hedonic wellbeing than the reference category (3). Controlling for wealth actually inflates the satisfaction levels of this group of people: given their low current wealth, these people are happier than the reference group, while other groups aren’t. This means that in England, people who have always been at the bottom of society throughout their life, feel more satisfied with life than those who have struggled to get higher up. One speculative interpretation of this finding is that being and remaining working class is socially acceptable in England, while in the U.S. a strong meritocratic attitude makes it harder to be satisfied in this position. The stronger support throughout the lifecycle for those at the bottom of the ladder in a welfare state such as the U.K. enables these higher levels of satisfaction.

### Social Mobility

**Fig. 2:**
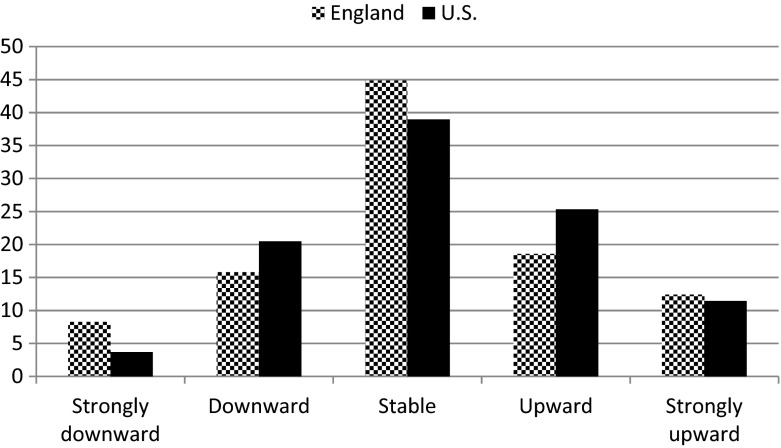
Distribution of social mobility (in %) in ELSA wave 2 (*n* = 6,269) and HRS wave 7 (*n* = 2,512) (2004)

To measure intergenerational social mobility we look at the difference between social origin and destination. A couple of noteworthy findings appear from the distributions by country (Fig. 2): there is more stability in England than in the U.S., and both upward and downward mobility in the U.S. tends to be more common. Descending two levels on the social ladder is more common in England. In summary, our data echo earlier findings of a higher, but not exceptionally higher, amount of social mobility in the U.S. compared to England.

The results of our analysis (Table 7) give moderate support to an influence of some types of social mobility on later life wellbeing. Downward mobility is detrimental to one’s level of satisfaction in both countries, over and above the influence of current wealth. Only in the U.S. upward mobility comes with a loss of satisfaction all other things being equal. This suggests that social rising can have unexpected repercussions, such as for example status anxiety, or feeling like an imposter who did not deserve this social promotion. Strongly upward mobility is beneficial to eudemonic wellbeing in England, and although the association is not significant when controlling for wealth it goes in the same direction in the U.S.

**Table 7:** Intergenerational social mobility influences on later life wellbeing

	England				U.S.			
	Hedonic (*n* = 6188)		Eudemonic (*n* = 5786)		Hedonic (*n* = 4779)		Eudemonic (*n* = 2263)	
	A	B	A	B	A	B	A	B
	B (SE) P	B (SE) P	B (SE) P	B (SE) P	B (SE) P	B (SE) P	B (SE) P	B (SE) P
Social mobility								
Strongly downward	−.601 (.297)*	−.477 (.296) ns	−0.541 (.328) ns	−.410 (.323) ns	−1.095 (.553)*	−.802 (.544) ns	−1.286 (.738) ns	−.869 (.722) ns
Downward	−432 (.211)*	−.547 (.210)**	−0.029 (.259) ns	−.235 (.255) ns	−.703 (.282)*	−.701 (.277)*	−.434 (.363) ns	−.273 (.355) ns
(ref. stable)								
Upward	−.006 (.205) ns	−.082 (.203) ns	0.395 (.241) ns	.200 (.278) ns	−.703 (.282)*	−.575 (.267)*	−.768 (.343)*	−.504 (.336) ns
Strongly upward	.139 (.236) ns	.019 (.235) ns	1.334 (.281) ***	.977 (.278)***	.143 (.365) ns	−.047 (.359) ns	.951 (.443)*	.684 (.434) ns
R square	.122	.134	.158	.185	.099	.130	.143	.184
Increase in R square compared to model without	.001	.001	.005	.003	.003	.002	.002	.000

Unstandardized regression coefficients of multivariate regression model (adjusted for (A) age, age^2^, gender, partnered, ethnic background, limitations in ADL and (B) + wealth quintile) *P*=<0.001 ***, *P*=<0.01 **, *P*=<0.05 *, *P*>0.05 ns

### Missing Value Analysis

As an important proportion of the early life course information is missing, we explicitly want to investigate the influence this could have on our analysis. In a first step we investigate if there is an association between being missing on a life course measure and the associated wellbeing score in each country, using a two sample *t* test (Table 8). This test investigates if there is a significant difference in the wellbeing scores of those who respond on a life course measure and those who do not.

**Table 8:** Results of *t* test investigating significance (at 95 % level) of differences in wellbeing scores of missing and responding group on life course measures

	England		US	
	Hedonic	Eudemonic	Hedonic	Eudemonic
Early life	Ns	Sig	Sig	Sig
Accumulation	Ns	Ns	Ns	Ns
Mobility	Ns	Sig	Sig	Sig

The results from our tests show that the wellbeing scores of people for whom we do not have information on their accumulation profile are not different for the different measures of wellbeing or the different countries. Missing information regarding early life and mobility does, however, seem to be associated with a a lower wellbeing score for three of the four tests in each case. A low level of education is the main determinant of missingness in both variables across both countries. It is reasonable to hypothesize that a low level of education is preceded by a middle or low socio-economic parental position, so that our results underestimate differences in relation to different early life positions. Also, a lower level of education in the context of social mobility relates to either a stable or upwardly mobile trajectory, which means the missing data, associated with lower wellbeing scores, could strengthen the negative effect of upward mobility in the US, and attenuate the positive association with strongly upward mobility.

## Conclusions

In this paper we investigate the influence of three life course mechanisms, critical period, accumulation and social mobility, on two dimensions of subjective wellbeing in later life, hedonic and eudemonic, in two societies, the U.S. and England.

Although earlier research (Blane et al. 2004; Jivraj and Nazroo 2014) has established the primacy of proximal influences on wellbeing, we illustrate that life course measures of socio-economic position add above and beyond current measures to explaining later life wellbeing. Early life seems to have the weakest and least consistent effect on wellbeing. Compared to coming from a modest family, growing up in a high socio-economic position has a moderate negative effect on hedonic wellbeing in England, but a strong positive effect on eudemonic wellbeing in the U.S. One obvious possibility for the divergence in results between countries is that we measured SEP in early life in different ways.

The idea that accumulation of advantage and disadvantage over the life course influences later life wellbeing receives strong support from our data, especially in the case of eudemonic wellbeing. In the U.S. we see a graded, dose–response association between life course exposure and autonomy, control and self-actualisation, while in England the association is less steeply graded but still present. Life satisfaction is positively related to less accumulation in the U.S., and there is an unexpected nonlinear relation in England: the group with the highest level of accumulations scores is significantly more satisfied than all other groups. This high score could result from the interplay between controlling for important current circumstances, such as health and wealth, and the tendency for people to give a moderately high satisfaction score, but equally could illustrate some kind of social acceptance, reflected in the welfare state, that it’s ok to stay working class.

There is some evidence for associations between social mobility and later life wellbeing, although not always in the expected direction. Downward intergenerational mobility has a clear negative effect on satisfaction with life both in the U.S. and England. In other words, people who end up in a lower socio economic position than their parents had, tend to be less satisfied with their life than those who have a stable trajectory. In England, those with a strongly upward social trajectory are better off in terms of eudemonic wellbeing than those that remain stable. A finding that goes against expectations, is that upwardly mobile are slightly less satisfied in the U.S. compared to those with stable trajectories. A first possible explanation questions the construct of social mobility for this specific cohort in the U.S.: The quality of work circumstances and job rewards in the U.S. service sector for an older worker who lost his manual job might not compare favourably, although formally it is social mobility. A second explanation is a form of status anxiety that accompanies a rise in social status. Conspicuous consumption by “new money” is one way to cope with this anxiety, but a negative impact on satisfaction could be a psychological effect of upward mobility. Overall, it is clear accumulation explains the highest share of variance across measures of wellbeing and across populations, which confirms our first hypothesis.

The two dimensions of wellbeing in later life clearly showed different relations with life course mechanisms. The association with satisfaction with life is often unclear or in a different direction than anticipated, which illustrates the need for more detailed information on expectations, value frames, and goals, which was not available in our data. Eudemonic wellbeing on the other hand is a more robust indicator of subjective quality of life, as it is clearly linked to accumulation and early life in both countries, and to social mobility in England. In general the added explanation of life course measures tended to be larger for eudemonic than for hedonic wellbeing, which confirms our second hypothesis. Two notable exceptions are the influence of social mobility in the US, and early life in England.

On the societal level, the major observation is that the life course has a larger influence in the U.S. than in England: unstandardized coefficients are larger, explained variances are larger and indicate larger differences in wellbeing in the U.S. than in England. This confirms that life course mechanisms are more closely associated with later life wellbeing in the U.S. than England, our third hypothesis. Furthermore, although there is more social mobility in the U.S., this social mobility seems relatively meaningless as it does not always result in higher wellbeing in later life.

This study is a secondary data analysis and suffers from a number of issues related to that. Firstly, we would ideally have socio-economic position measured identically in both surveys across more time points. As this was not possible, discrepancies between time points and surveys might substantially influence our results. Secondly, we are taking a bird’s eye view on the life course, focusing on relatively rough classifications, direct associations and simple methods of analysis. Thirdly, the significant amount of missing values in the life course measures means that we could be underestimating early life influence, or that having a stable or upwardly mobile trajectory is less positive for wellbeing than we found. We are fully aware of these limitations, but equally believe in the principle of Ockham’s razor, and wanted to test associations in a simple and straightforward matter, strengthened by a comparative perspective.
